# Injection of hydrogel spacer increased maximal intrafractional prostate motion in anterior and superior directions during volumetric modulated arc therapy-stereotactic body radiation therapy for prostate cancer

**DOI:** 10.1186/s13014-022-02008-3

**Published:** 2022-02-23

**Authors:** Subaru Sawayanagi, Hideomi Yamashita, Mami Ogita, Ryosuke Takenaka, Yuki Nozawa, Yuichi Watanabe, Toshikazu Imae, Osamu Abe

**Affiliations:** Department of Radiology, University of Tokyo Hospital, 7-3-1, Hongo, Bunkyo-ku, Tokyo, 113-8655 Japan

**Keywords:** Prostate cancer, Stereotactic body radiation therapy, Intrafractional prostate motion, Transperineal ultrasound, Hydrogel spacer

## Abstract

**Background:**

The aim of this study was to clarify the association between intrafractional prostate shift and hydrogel spacer.

**Methods:**

Thirty-eight patients who received definitive volumetric modulated arc therapy (VMAT)-stereotactic body radiation therapy (SBRT) for prostate cancer with prostate motion monitoring in our institution in 2018–2019 were retrospectively evaluated. In order to move the rectum away from the prostate, hydrogel spacer (SpaceOAR system, Boston Scientific, Marlborough, the United States) injection was proposed to the patients as an option in case of meeting the indication of use. We monitored intrafractional prostate motion by using a 4-dimensional (4D) transperineal ultrasound device: the Clarity 4D ultrasound system (Elekta AB). The deviation of the prostate was monitored in each direction: superior-inferior, left–right, and anterior–posterior. We also calculated the vector length. The maximum intrafractional displacement (MID) per fraction for each direction was detected and mean of MIDs was calculated per patient. The MIDs in the non-spacer group and the spacer group were compared using the unpaired t-test.

**Results:**

We reviewed 33 fractions in eight patients as the spacer group and 148 fractions in 30 patients as the non-spacer group. The superior MID was 0.47 ± 0.07 (mean ± SE) mm versus 0.97 ± 0.24 mm (*P* = 0.014), the inferior MID was 1.07 ± 0.11 mm versus 1.03 ± 0.25 mm (*P* = 0.88), the left MID was 0.74 ± 0.08 mm versus 0.87 ± 0.27 mm (*P* = 0.55), the right MID was 0.67 ± 0.08 mm versus 0.92 ± 0.21 mm (*P* = 0.17), the anterior MID was 0.45 ± 0.06 mm versus 1.16 ± 0.35 mm (*P* = 0.0023), and the posterior MID was 1.57 ± 0.17 mm versus 1.37 ± 0.22 mm (*P* = 0.56) in the non-spacer group and the spacer group, respectively. The max of VL was 2.24 ± 0.19 mm versus 2.89 ± 0.62 mm (*P* = 0.19), respectively.

**Conclusions:**

Our findings suggest that maximum intrafractional prostate motion during VMAT-SBRT was larger in patients with hydrogel spacer injection in the superior and anterior directions. Since this difference seemed not to disturb the dosimetric advantage of the hydrogel spacer, we do not recommend routine avoidance of the hydrogel spacer use.

## Background

External beam radiation therapy (EBRT) is recognized as one of the primary treatment options for patients with prostate cancer (PCa) [1, 2]. Intensity modulated radiation therapy (IMRT) with image-guided radiation therapy (IGRT) technique is currently the gold standard for EBRT. A low α/β ratio of PCa has encouraged hypofractionation and stereotactic body radiation therapy (SBRT), or extreme hypofractionation, which is currently considered as a promising option of EBRT [3–6]. Recently, the trial of the single-fraction SBRT for localized prostate cancer under the intrafractional tracking was reported [7]. While higher quality of positioning is required in SBRT to optimize treatments, it is known that organs in pelvis including prostate are shifting under the influence of rectal volume, bladder volume, and change of muscle tension among other things [8–17]. These intrafractional uncertainties possibly affect the dose distributions [18–20].

Less is known about risk factors related to intrafractional prostate motion. While the variability in location of external skin markers relative to internal anatomy in obese patients caused a significant difference in terms of interfractional prostate shift [21], there was no relationship between body mass index (BMI) and intrafractional prostate motion [22]. It was reported that the shorter the maximum rectal diameter is, the less the intrafractional prostate motion is [23].

The injection of hydrogel spacer between the prostate and the rectum has been introduced for RT for PCa to separate the prostate from the anterior wall of the rectum, which contributes to reducing the RT dose of the rectum and reduces gastrointestinal and genitourinary toxicities [24–27]. On the other hand, the complications related to the injection of hydrogel spacer have been reported [27, 28]. Regarding prostate motion, it was shown that the insertion of hydrogel spacer did not greatly limit interfractional and intrafractional prostate displacements [29–33]. We hypothesized that the hydrogel spacer potentially implicates the prostate variability during RT. The aim of this study was to clarify the association between the intrafractional prostate shift and the hydrogel spacer.

## Methods

A total of 38 patients with histologically confirmed prostate cancer who received definitive volumetric modulated arc therapy (VMAT)-SBRT with prostate motion monitoring with or without androgen deprivation therapy (ADT) for PCa in our institution in 2018–2019 were retrospectively evaluated. The study was reviewed and approved by the institutional review board and ethics committee. Examination number was 3372.

### Radiotherapy

A total dose of 36.25–40 Gy in five fractions was prescribed to 95% of the planning target volume (PTV) every other weekday. All patients received CT scans which were reconstructed 1-mm-thick slices with a full bladder for treatment planning. A rectal enema was prescribed before simulation and before each treatment session to empty the contents of the rectum. The clinical target volume (CTV) consisted of prostate with or without seminal vesicles according to the risk classification of the NCCN guidelines version 1.2018. The CTV was extended by 5 mm in every direction except posterior with 3 mm extension to generate the PTV. We used Monaco (Elekta AB, Stockholm, Sweden) as the treatment planning system. In order to move the rectum away from the prostate, the hydrogel spacer (SpaceOAR system, Boston Scientific, Marlborough, the United States) was transperineally injected into the recto-prostatic space of patients who hoped to receive it in case of meeting the indication of use. KV cone beam CT (CBCT) scans were acquired after the setup before each treatment session to reduce the interfraction error of patient positioning.

### Motion monitoring

Intrafraction motion of the prostate was monitored by a 4-dimensional (4D) transperineal ultrasound (US) device: the Clarity 4D ultrasound system (Elekta AB) with an autoscanning perineal US probe. We regarded the prostate position when CBCT scans finished as the baseline position. Monitoring time was defined as the time from the end of CBCT to the end of radiation. We verified these time points with the logs in the Clarity system. The deviation of the prostate from the baseline position was monitored as a function of time along the three directions: superior-inferior (SI), left–right (LR), and anterior–posterior (AP). We also calculated the vector length (VL) at each point in time.

Large spike-like prostate displacement was seen during couch shift for patient position adjustment in some fractions though the prostate position we evaluated was relative to the couch position. These spike-like displacements continued for about 10 s. We excluded these displacements from analyses because all patients immediately recovered from this error before the start of radiation. The maximum intrafractional displacement (MID), the mean intrafractional displacement (mID), and the 95th percentile maximum intrafractional displacement (95th-MID) per fraction for each direction was detected and mean of them was calculated per patient. The 95th-MID was the value within which the prostate displacement was during 95% of the monitoring time. We chose these objects for our study for the following reasons. Ignoring the interfractional errors, in other words, hypothesizing that we can perfectly bring the patient to the treatment position using CBCT, the MID for each direction was the margin itself we needed to add to the CTV in order to include all the errors into the irradiated field of the single treatment. To exclude the extreme movement in a very short time, we also analyzed the 95th-MID. We can recognize which direction and how far the prostate tends to move during the monitoring time by the mIDs.

The Stroom formula [34] (= 2.0 Σ + 0.7 σ) and the van Herk formula [35] (= 2.5 Σ + 0.7 σ) were used to calculate the CTV-PTV margin derived from intrafractional error, where Σ is the systematic error and σ is the random error.

### Statistical analysis

Differences in characteristic variables between patients without spacer and those with spacer were tested using the χ^2^-test for categorical variables and the unpaired t-test for continuous variables. In this study, the volume from the slice 1 cm above the highest part of the PTV to the slice 1 cm below the lowest part of the PTV in axial slices was considered as rectum volume. Bladder volume in this context included internal urine. The MIDs, the mIDs, and the 95th-MIDs in the non-spacer group and the spacer group were compared using the unpaired t-test. Multiple regression analysis was used to detect risk factors related to the MID. All statistical analyses were two-sided and performed using R, version 4.0.3. Results were considered statistically significant at *P* < 0.05.

## Results

### Patient characteristics

We reviewed 33 fractions in eight patients as the spacer group and 148 fractions in 30 patients as the non-spacer group. The baseline patient characteristics were presented in Table 1. There was no statistically significant difference between the two groups except for rectal volume. The median age was 73 (58–85) and 79 (66–84) years (*P* = 0.22) and the mean monitoring time was 275 ± 42 (mean ± SD) and 279 ± 33 s (*P* = 0.82) in the non-spacer group and the spacer group, respectively. Twenty-one patients (70.0%) in the non-spacer group and six patients (75.0%) in the spacer group received neoadjuvant ADT (*P* = 0.99), respectively. The mean BMI was 23.5 ± 2.7 and 23.6 ± 3.4 kg/m^2^ in the non-spacer group and the spacer group, respectively (*P* = 0.93). Nine patients (30.0%) in the non-spacer and one patient (12.5%) in the spacer group underwent abdominal surgery before RT, respectively (*P* = 0.65). The mean volume of prostate (non-spacer vs. spacer group, 31.1 ± 12.4 cc vs. 25.0 ± 9.2 cc, *P* = 0.22) and bladder (277.1 ± 169.2 cc vs. 205.2 ± 77.9 cc, *P* = 0.26) measured on planning CT in each group was not significantly different. The rectum volume on planning CT in the non-spacer group was larger than the spacer group (55.4 ± 15.5 cc vs. 43.4 ± 9.0 cc, *P* = 0.047).

**Table 1:** Baseline patient characteristics

Parameter	Non-spacer group	Spacer group	*P* value
	*N* = 30	*N* = 8	
Age, years, median (range)	73 (58–85)	79 (66–84)	0.22^b^
Body mass index, mean ± SD	23.5 ± 2.7	23.6 ± 3.4	0.93^b^
Clinical T stage, n (%)			0.78^c^
1c	6 (20%)	3 (37.5%)	
2a	13 (43.3%)	2 (25.0%)	
2b	2 (6.7%)	1 (12.5%)	
2c	5 (16.7%)	2 (25.0%)	
3a	1 (3.3%)	0 (0%)	
3b	2 (6.7%)	0 (0%)	
4	1 (3.3%)	0 (0%)	
Mean monitoring time, seconds, mean ± SD	275 ± 42	279 ± 33	0.82^b^
Neoadjuvant ADT, n (%)			0.99^c^
No	9 (30.0%)	2 (25.0%)	
Yes	21 (70.0%)	6 (75.0%)	
History of abdominal surgery, n (%)			0.65^c^
No	21 (70.0%)	7 (87.5%)	
Yes	9 (30.0%)	1 (12.5%)	
Prostate volume*, cc, mean ± SD	31.1 ± 12.4	25.0 ± 9.2	0.22^b^
Rectum volume*^a^, cc, mean ± SD	55.4 ± 15.5	43.4 ± 9.0	0.047^b†^
Bladder volume*, cc, mean ± SD	277.1 ± 169.2	205.1 ± 77.9	0.26^b^

Abbreviations: *ADT* androgen deprivation therapy, *SD* standard deviation

*Measured on planning CT

^a^From the slice 1 cm above the PTV to the slice 1 cm below the PTV in axial slices

^b^By unpaired t-test

^c^By χ^2^-test

^†^Statistically significant

### Motion analysis

**Fig. 1:**
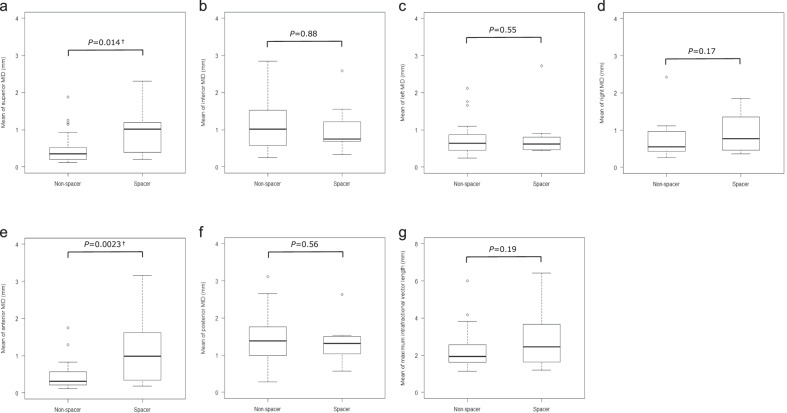
Boxplots of the maximum intrafractional displacement. **a** Mean of superior MID. **b** Mean of inferior MID. **c** Mean of left MID. **d** Mean of right MID. **e** Mean of anterior MID. **f** Mean of posterior MID. **g** Mean of maximum intrafractional vector length. *Abbreviations: MID* maximum intrafracitonal displacement. ^†^Statistically significant

The comparison of MID, mID, and 95th-MID for each direction and the VL between the two groups was presented in Table 2 and the boxplots of the MIDs were shown in Fig. 1. The superior MID was 0.47 ± 0.07 (mean ± SE) mm versus 0.97 ± 0.24 mm (*P* = 0.014), the inferior MID was 1.07 ± 0.11 mm versus 1.03 ± 0.25 mm (*P* = 0.88), the left MID was 0.74 ± 0.08 mm versus 0.87 ± 0.27 mm (*P* = 0.55), the right MID was 0.67 ± 0.08 mm versus 0.92 ± 0.21 mm (*P* = 0.17), the anterior MID was 0.45 ± 0.06 mm versus 1.16 ± 0.35 mm (*P* = 0.0023), the posterior MID was 1.57 ± 0.17 mm versus 1.37 ± 0.22 mm (*P* = 0.56), and the MID as VL was 2.24 ± 0.19 mm versus 2.89 ± 0.62 mm (*P* = 0.19) in the non-spacer group and the spacer group, respectively. The superior-inferior mID was − 0.34 ± 0.06 mm versus − 0.12 ± 0.19 mm (*P* = 0.16), the left–right mID was 0.02 ± 0.04 mm versus 0.04 ± 0.25 mm (*P* = 0.93), the anterior–posterior mID was − 0.55 ± 0.09 mm versus − 0.21 ± 0.27 mm (*P* = 0.13), the mID as VL was 1.05 ± 0.10 mm versus 1.56 ± 0.43 mm (*P* = 0.10) in the non-spacer group and the spacer group, respectively. The prostate tended to move caudally and posteriorly with or without the spacer. The superior 95th-MID was 0.27 ± 0.05 mm versus 0.74 ± 0.19 mm (*P* = 0.0029), the inferior 95th-MID was 0.87 ± 0.09 mm versus 0.83 ± 0.25 mm (*P* = 0.87), the left 95th-MID was 0.51 ± 0.07 mm versus 0.61 ± 0.24 mm (*P* = 0.60), the right 95th-MID was 0.41 ± 0.06 mm versus 0.64 ± 0.18 mm (*P* = 0.17), the anterior 95th-MID was 0.23 ± 0.04 mm versus 0.85 ± 0.31 mm (*P* = 0.0020), the posterior 95th-MID was 1.31 ± 0.16 mm versus 1.05 ± 0.14 mm (*P* = 0.42), and the 95th-MID as VL was 1.89 ± 0.18 mm versus 2.51 ± 0.55 mm (*P* = 0.19) in the non-spacer group and the spacer group, respectively. The superior and anterior MIDs and 95th-MIDs were smaller in the non-spacer group, while the other MIDs, mIDs, and 95th-MIDs were not significantly different in the two groups.

**Table 2:** Mean of the intrafractional displacements per patient

Direction	Non-spacer group	Spacer group	*P* value^a^
	*N* = 30	*N* = 8	
	mm, Mean ± SE	mm, Mean ± SE	
Maximum intrafractional displacements			
Superior	0.47 ± 0.07	0.97 ± 0.24	0.014^†^
Inferior	1.07 ± 0.11	1.03 ± 0.25	0.88
Left	0.74 ± 0.08	0.87 ± 0.27	0.55
Right	0.67 ± 0.08	0.92 ± 0.21	0.17
Anterior	0.45 ± 0.06	1.16 ± 0.35	0.0023^†^
Posterior	1.57 ± 0.17	1.37 ± 0.22	0.56
Vector length	2.24 ± 0.19	2.89 ± 0.62	0.19
Mean intrafracitonal displacements^b^			
Superior-inferior	− 0.34 ± 0.06	− 0.12 ± 0.19	0.16
Left–right	0.02 ± 0.04	0.04 ± 0.25	0.93
Anterior–posterior	− 0.55 ± 0.09	− 0.21 ± 0.27	0.13
Vector length	1.05 ± 0.10	1.56 ± 0.43	0.10
95th percentile intrafractional displacements			
Superior	0.27 ± 0.05	0.74 ± 0.19	0.0029^†^
Inferior	0.87 ± 0.09	0.83 ± 0.25	0.87
Left	0.51 ± 0.07	0.61 ± 0.24	0.60
Right	0.41 ± 0.06	0.64 ± 0.18	0.17
Anterior	0.23 ± 0.04	0.85 ± 0.31	0.0020^†^
Posterior	1.31 ± 0.16	1.05 ± 0.14	0.42
Vector length	1.89 ± 0.18	2.51 ± 0.55	0.19

Abbreviations: *SE* standard error

^a^By unpaired t-test

^b^Superior, left, and anterior displacements were written as positive number

^†^Statistically significant

### Multivariate analysis

The results of multivariate analyses for MIDs for each direction were shown in Table 3. We included age, spacer injection, rectum volume, and the duration of monitoring as explanatory variables. Spacer injection was the independent risk factor of superior and anterior MIDs. There was no independent risk factor of inferior, left, right, and posterior MIDs and maximum VL.

**Table 3:** Multivariate analyses for maximum intrafractional displacement

	Regression coefficient (95% CI)	*P* value^a^
Superior MID		
Age (continuous)	− 0.012 (− 0.036–0.012) per year	0.31
Spacer injection		0.014^†^
Non-spacer	Reference	
Spacer	0.546 (0.118–0.975)	
Rectum volume (continuous)	0.001 (− 0.001–0.013) per cc	0.80
Monitoring time (continuous)	0.001 (− 0.003–0.005) per second	0.66
Inferior MID		
Age (continuous)	0.026 (− 0.003–0.055) per year	0.082
Spacer injection		0.31
Non-spacer	Reference	
Spacer	− 0.263 (− 0.783–0.256)	
Rectum volume (continuous)	− 0.011 (− 0.025–0.002) per cc	0.10
Monitoring time (continuous)	0.000 (− 0.005–0.005) per second	0.99
Left MID		
Age (continuous)	0.011 (− 0.015–0.038) per year	0.39
Spacer injection		0.70
Non-spacer	Reference	
Spacer	0.089 (− 0.382–0.560)	
Rectum volume (continuous)	0.000 (− 0.012–0.013) per cc	0.98
Monitoring time (continuous)	0.000 (− 0.005–0.004) per second	0.84
Right MID		
Age (continuous)	0.008 (− 0.015–0.031) per year	0.48
Spacer injection		0.23
Non-spacer	Reference	
Spacer	0.245 (− 0.161–0.652)	
Rectum volume (continuous)	0.002 (− 0.008–0.013) per cc	0.67
Monitoring time (continuous)	0.002 (− 0.001–0.006) per second	0.22
Anterior MID		
Age (continuous)	0.008 (− 0.020–0.036) per year	0.57
Spacer injection		0.0074^†^
Non-spacer	Reference	
Spacer	0.692 (0.199–1.185)	
Rectum volume (continuous)	0.001 (− 0.012–0.014) per cc	0.84
Monitoring time (continuous)	0.002 (− 0.003–0.006) per second	0.50
Posterior MID		
Age (continuous)	0.004 (− 0.042–0.049) per year	0.87
Spacer injection		0.35
Non-spacer	Reference	
Spacer	− 0.375 (− 1.174–0.424)	
Rectum volume (continuous)	− 0.012 (− 0.033–0.009) per cc	0.24
Monitoring time (continuous)	0.002 (− 0.006–0.009) per second	0.65
Maximum intrafractional vector length		
Age (continuous)	0.023 (− 0.040–0.085) per year	0.46
Spacer injection		0.42
Non-spacer	Reference	
Spacer	0.441 (− 0.664–1.547)	
Rectum volume (continuous)	− 0.010 (− 0.039–0.019) per cc	0.48
Monitoring time (continuous)	0.002 (− 0.008–0.012) per second	0.69

Abbreviations: *MID* maximum intrafracitonal displacement, *CI* confidence interval

^a^By multiple regression analysis

^†^Statistically significant

### Margin calculation

The mean (μ), the systematic error (Σ), and the random error (σ) of intrafractional shift of the prostate were μ = (− 0.43, 0.06, − 1.12) mm, Σ = (0.91, 0.78, 1.17) mm, and σ = (1.24, 1.25, 1.86) mm in the SI, LR, and AP directions, respectively in the non-spacer group. In the spacer group, μ = (− 0.79, 0.18, − 0.71) mm, Σ = (1.12, 1.01, 1.32) mm and σ = (2.12, 1.21, 2.42) mm, respectively. According to the Stroom formula, margins (M) were as follows: M = (2.69, 2.42, 3.64) mm in the SI, LR, and AP directions in the non-spacer group, and M = (3.71, 2.87, 4.34) mm in the spacer group. According to the van Herk formula, M = (3.14, 2.81, 4.23) mm in the non-spacer group and M = (4.27, 3.37, 5.00) mm in the spacer group, respectively.

## Discussion

Nowadays, hydrogel spacer is widely used for the radiotherapy for prostate cancer. It has been shown that the hydrogel spacer improves the dose distribution and reduces gastrointestinal and genitourinary toxicities [24–27], while some complications related to the injection were observed [27, 28]. They included anaphylaxis, colostomy, pulmonary embolism, and prostatic abscess. Routine use of the hydrogel spacer is still controversial.

We investigated the intrafractional prostate motion during VMAT-SBRT for PCa. Considering that SE of the MID, the mID, and the 95th-MID was small enough in each direction, these outcomes seemed to be correctly averaged. Calculated margins according to the Stroom formula were larger in the spacer group than the non-spacer group by (1.02, 0.45, 0.70) mm in the SI, LR, and AP directions, respectively. Based on the van Herk formula, calculated margins were larger in the spacer group than the non-spacer group by (1.13, 0.56, 0.77) mm in the SI, LR, and AP directions, respectively.

Errors related to the quality of radiotherapy are categorized into interfractional errors and intrafractional errors. While interfractional errors can be minimized by using high-precision techniques of patient positioning, intrafractional errors caused by organ motion depended on the patient himself and are difficult to be controlled.

Development of monitoring devices has contributed to knowing the intrafrational organ motions. Mah et al. [8] investigated the intrafractional prostate motion of 42 patients with prostate cancer using cine-MRI. They reported that the displacements of prostate (mean ± SD) were 0.0 ± 3.4 mm, 0.0 ± 1.5 mm, 0.2 ± 2.9 mm in the SI, LR, and AP dimensions, respectively. Willoughby et al. [12] used the Calypso 4D localization system which is real-time tracking system with implanted electromagnetic transponders to track the intrafractional shift of prostate. They showed that the average (± SD) of the maximum differences in 11 cases were 3.61 ± 3.13 mm, 0.91 ± 0.35 mm, 3.92 ± 4.32 mm in the SI, LR, and AP directions, respectively. Pinkawa et al. [14] demonstrated that the intrafractional displacements of prostate (mean ± SD) were 0.0 ± 2.0 mm, 0.2 ± 1.9 mm, 0.6 ± 2.2 mm in the SI, LR, and AP directions in 32 patients with prostate cancer by using transabdominal US tracking system. Comparable level of the intrafractional prostate motion with these studies was seen in our study. The average (± SE) of the maximum vector displacement was 2.24 ± 0.19 mm and 2.89 ± 0.62 mm in the non-spacer and the spacer group, respectively.

Sihono et al. [15] suggested the patient population-based margin according to the van Herk formula is as follows: 1.10 mm, 1.25 mm, and 1.33 mm in the SI, LR, and AP directions, respectively. They used the Clarity system just like our study. We demonstrated the larger margins calculated based on our population; 3.14 mm, 2.81 mm, 4.23 mm in the non-spacer group and 4.27 mm, 3.37 mm, 5.00 mm in the spacer group. The difference is probably ascribed to the fact that Sihono et al. may have used mean intrafractional motion for margin calculation, whereas we used maximum intrafractional motion. The mean intrafractional prostate displacement by Richter et al. [16], who also used the Clarity system, was remarkably little: − 0.06 ± 0.49 mm, − 0.09 ± 0.61 mm, and − 0.01 ± 0.78 mm in the SI, LR and AP directions, respectively. Levin-Epstein et al. [17] reported that the margins calculated with the intra-fractional motion were 2.7 mm, 1.9 mm, and 3.1 mm in the SI, LR, and AP directions, respectively.

**Fig. 2:**
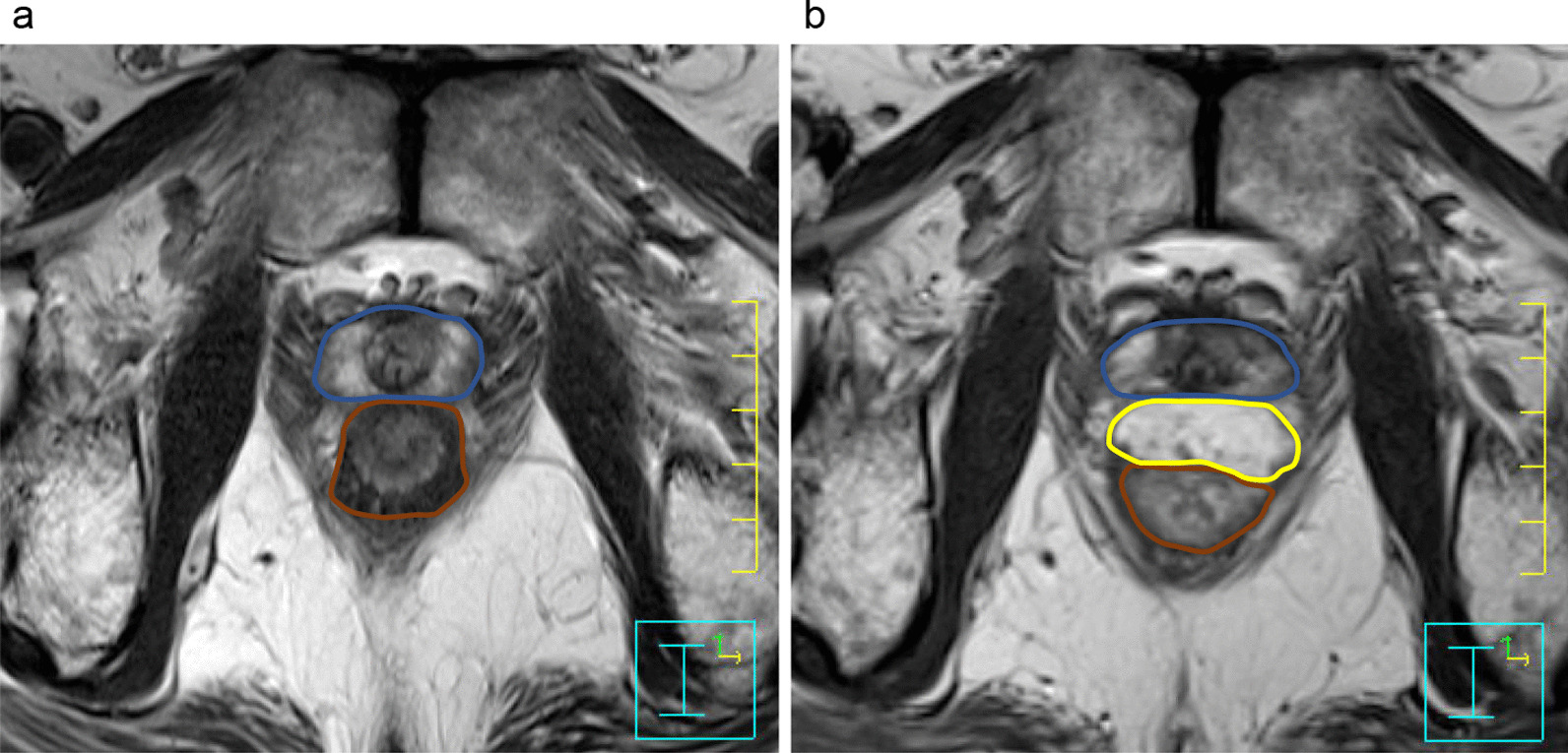
MRI images of the same patient. **a** The image before spacer injection. **b** The image after spacer injection. Prostate (blue), rectum (brown), and spacer (yellow) are shown

Knowledge about parameters related to intrafractional prostate motion is absolutely limited. Brown et al. [22] showed that there was no statistically significant relationship between intrafractional prostate motion and BMI by using linear regression analysis. Oates et al. [23] investigated a relationship between maximum rectal diameter (MRD) and intrafractional prostate motion. They showed with 90% confidence that for a MRD ≤ 3 cm, prostate displacement will be ≤ 5 mm and that for a MRD ≤ 3.5 cm, prostate displacement will be ≤ 5.5 mm. By prescribing a rectal enema and performing CBCT before each treatment session, the variety of MRD may have been minimized in our study. Rectum volume was smaller in the spacer group, which may be caused by the deformation of rectum by the pressure from the anterior direction by the injected hydrogel spacer (Fig. 2). However, rectum volume was not an independent risk factor for prostate displacement in the multivariate analysis. The displacement of prostate was shown to be smaller in step-and-shoot IMRT fractions than in VMAT fractions due to the shorter treatment time of VMAT by Ballhausen et al. [36]. In the present study, we treated all patients with VMAT using flattening filter free (FFF) beams and monitoring time from the end of CBCT to the end of radiation was about 4.5 min. According to our study, monitoring time did not significantly affect prostate shift.

Picardi et al. [30] showed that hydrogel spacer injection into the recto-prostatic space did not significantly influence the interfraction prostate motion based on the analysis using implanted fiducial markers and CBCT. It was reported that hydrogel spacer insertion significantly reduced the intrafraction rotational shift in the AP direction on cine-MRI by Cuccia et al. [31] and they concluded that hydrogel spacer contributed to limiting prostate intrafractional motion. On the other hand, Juneja et al. [29] showed that the average of the mean intrafractional vector displacement of prostate was significantly larger in patients with hydrogel spacer than those without spacer by analyzing the implanted electromagnetic markers position on kV fluoroscopy. The difference between the two groups was 0.4 mm on their study. In our study, there was no significant difference in maximum VL, whereas superior and anterior MIDs were significantly larger in the spacer group in our study, and the difference between the two groups were 0.5 mm in the superior direction and 0.7 mm in the anterior direction. Suzuki et al. [32] also reported the effect of the hydrogel spacer on the intrafractional prostate motion during the CyberKnife treatment for prostate cancer. They calculated the mean intrafractional prostate motion in the SI, LR, and AP directions for each patient using two fiducial markers and kV X-ray images and compared the results of the spacer group with the non-spacer group. There was no statistically significant difference between the two groups in any directions.

The present study was associated with some limitations. One of the major limitations was the fact that we did not investigate the effect of the intrafractional prostate motion on the dosimetric and clinical outcome. Li et al. [37] suggested an analytical model for the estimation of respiratory motion-induced dose uncertainty in spot-scanning proton beam therapy. Kontaxis et al. [20] quantified the delivered dose for the prostate cancer treated with the MR-linac based on the 3D cine-MRI and the treatment log files. They reported that the average drop from the planned dose in D99% coverage for the CTV was 2.1% ± 2.9%. In our study, the hydrogel spacer increased the superior and anterior MID of the prostate, which may have increased the dose delivered to the area inferior and posterior to the prostate. Because the difference of the MID between the two groups was 0.5–0.7 mm, the dose to the anterior wall of the rectum in the spacer group seems not to have increased due to the recto-prostatic space spread by the spacer. Considering the dosimetric and clinical merit of the hydrogel spacer [24–27], our results do not support routine avoidance of the hydrogel spacer use.

Another limitation of our study was the fact that the quality of our results depends on the accuracy of the Clarity system. The Clarity system has the advantage of not requiring fiducial markers implantation or additional hardware installation and no radiation exposure [38]. The disadvantage of the Clarity system is operator dependence [15]. In our study, every operator had received the training of this system in advance. Zhou et al. [39] investigated the discrepancy between the Clarity system and CBCT as the positioning device. The average (± SD) discrepancies were − 0.03 ± 5.22 mm, 0.18 ± 2.87 mm, and 0.31 ± 4.37 mm in the SI, LR, and AP directions, respectively when the Clarity was transperineally used. Grimwood et al. [38] reported that the median difference between Clarity-defined intrafractional marker locations and portal-imaged marker locations was 0.6 mm. Biston et al. [40] also reported that the mean differences between intrafractional displacements observed on patients with the Clarity system and the intraprostatic electromagnetic transmitters were ≤ 0.55 mm in all directions except for one patient.

The other limitation was small sample size, in particular the spacer group. Most characteristics were similar between the two groups, but the rectum volume was smaller in the spacer group, which may be due to accidental bias. However, rectum volume was not the independent risk factor of the MIDs according to the multivariate analysis.

## Conclusions

Our findings suggest that maximum intrafractional prostate motion during VMAT-SBRT was larger in patients with hydrogel spacer injection in the superior and anterior directions. Since this difference seemed not to disturb the dosimetric advantage of the hydrogel spacer, we do not recommend routine avoidance of the hydrogel spacer use. Further studies are expected in order to clarify the cause that prostate in patients with hydrogel spacer tends to move during RT, and to find other factors related to intrafractional prostate motion.

## Data Availability

The datasets used and/or analyzed during the current study are available from the corresponding author on reasonable request.

## Abbreviations

ADT: Androgen deprivation therapy
AP: Anterior–posterior
BMI: Body mass index
CBCT: Cone beam computed tomography
CT: Computed tomography
CTV: Clinical target volume
EBRT: External beam radiation therapy
FFF: Flattening filter free
IGRT: Image-guided radiation therapy
IMRT: Intensity modulated radiation therapy
LR: Left–right
mID: Mean intrafractional displacement
MID: Maximum intrafractional displacement
MRD: Maximum rectal diameter
MRI: Magnetic resonance imaging
NCCN: National Comprehensive Cancer Network
PTV: Planning target volume
RT: Radiotherapy
SBRT: Stereotactic body radiation therapy
SD: Standard deviation
SE: Standard error
SI: Superior-inferior
US: Ultrasound
VL: Vector length
VMAT: Volumetric modulated arc therapy
95th-MID: 95Th percentile maximum intrafractional displacement
